# New classification of oesophageal and gastric carcinomas derived from changing patterns in epidemiology

**DOI:** 10.1038/sj.bjc.6690429

**Published:** 1999-05

**Authors:** K Dolan, R Sutton, S J Walker, A I Morris, F Campbell, E M I Williams

**Affiliations:** Departments of Surgery, Gastroenterology and Pathology, Royal Liverpool University Hospital, Daulby Street, Liverpool, L69 36A, UK; Department of Public Health, University of Liverpool, Liverpool, UK

**Keywords:** oesophageal carcinoma, gastric carcinoma, epidemiology, classification

## Abstract

The current ICD-O classification of carcinomas of the oesophagus and stomach causes epidemiological and clinical confusion. This study compares the epidemiological and clinical features of each subtype and subsite of adenocarcinomas of the oesophagus and stomach, to assess requirements for a new classification of these carcinomas. Data were extracted with appropriate validity checks on all cases of oesophageal and gastric carcinomas identified throughout the period 1974–1993 by the Merseyside and Cheshire Cancer Registry, which covers a population of 2.5 million. The incidence of adenocarcinomas of the lower oesophagus and cardia trebled in males, and doubled in females, whereas adenocarcinoma of the subcardia region of the stomach declined in both sexes. Adenocarcinomas of the lower oesophagus and of the cardia were similar for median age at diagnosis, male to female ratio, percentage of patients who smoked and survival; both were significantly different from carcinomas of the subcardia in these respects. These data imply that adenocarcinomas of the lower oesophagus and cardia are the same disease. A new subsite classification of oesophageal and gastric carcinomas is proposed that includes the gastro-oesophageal junction as a distinct subsite, to facilitate surveillance, management and research. © 1999 Cancer Research Campaign


*British Journal of Cancer* (1999) **80**(5/6), 834–842
© 1999 Cancer Research Campaign
Article no. bjoc.1998.0429

### New classification of oesophageal and gastric carcinomas derived from changing patterns in epidemiology

**K Dolan[1], R Sutton[1], SJ Walker[1], AI Morris[2], F Campbell[3] and EMI Williams[4]**

Departments of [1]Surgery, [2]Gastroenterology and [3]Pathology, Royal Liverpool University Hospital, Daulby Street, Liverpool L69 36A, UK; [4]Department of Public Health, University of Liverpool, Liverpool, UK

**Summary** The current ICD-O classification of carcinomas of the oesophagus and stomach causes epidemiological and clinical confusion. This study compares the epidemiological and clinical features of each subtype and subsite of adenocarcinomas of the oesophagus and stomach, to assess requirements for a new classification of these carcinomas. Data were extracted with appropriate validity checks on all cases of oesophageal and gastric carcinomas identified throughout the period 1974–1993 by the Merseyside and Cheshire Cancer Registry, which covers a population of 2.5 million. The incidence of adenocarcinomas of the lower oesophagus and cardia trebled in males, and doubled in females, whereas adenocarcinoma of the subcardia region of the stomach declined in both sexes. Adenocarcinomas of the lower oesophagus and of the cardia were similar for median age at diagnosis, male to female ratio, percentage of patients who smoked and survival; both were significantly different from carcinomas of the subcardia in these respects. These data imply that adenocarcinomas of the lower oesophagus and cardia are the same disease. A new subsite classification of oesophageal and gastric carcinomas is proposed that includes the gastro-oesophageal junction as a distinct subsite, to facilitate surveillance, management and research.

**Keywords:** oesophageal carcinoma; gastric carcinoma; epidemiology; classification

Adenocarcinoma of the oesophagus is increasing in a number of regions throughout the world, whilst the incidence of gastric carcinoma is decreasing (Blot et al, 1991; McKinney et al, 1995; Thomas et al, 1996; Hansen et al, 1997). Indeed, it has been claimed that the ratio of oesophageal to gastric adenocarcinoma has increased from 1:40 in the 1940s to 1:4 in the 1980s (Locke et al, 1995). Despite the apparent overall decline in carcinoma of the stomach, increases in carcinoma of the proximal stomach (Craanen et al, 1992) and of the gastric cardia (Powell and McConkey, 1990) have been reported. Although these increases appear contradictory, the cardia is that part of the stomach closest to and conjoined with the oesophagus. It is therefore possible that adenocarcinoma of the oesophagus and of the gastric cardia may represent the same disease, and the classification of adenocarcinomas of the cardia as gastric carcinomas may be misleading. We addressed this issue by detailed examination of changes in the epidemiological and clinical features of oesophageal and gastric carcinoma occurring in a single population of 2.5 million over a 20-year period.

### MATERIALS AND METHODS

### Identification and classification of cases

The Merseyside and Cheshire Cancer Registry (MCCR) provided clinical and pathological details on all registered cases of primary oesophageal and gastric carcinoma occurring in local residents between 1974 and 1993.

The ICD-O classification (version 2; Percy et al, 1990) was used to subdivide carcinomas by subtype and subsite, but is unsatisfactory in the distal stomach, where some subsites overlap. Therefore in this study the ICD-O classification for gastric tumours was simplified into cardia, fundus and subfundus groups as shown in Table 1.

### Data verification

MCCR data derive from pathology reports, hospital case notes and death certificates, and are checked by registration officers before being entered onto a computerized database. Additionally, systematic validity checks were performed by one of the authors (KD) through review of abstracted hospital case notes and pathology reports, as follows. Subtype and subsite classification checks were performed on 100 cases (2%) of oesophageal carcinoma and 200 cases (2%) of gastric carcinoma randomly (every 50th case) selected from tables compiled by subtype or subsite. The oesophageal sample was stratified by subtype and comprised 25 cases each of adenocarcinoma and squamous cell carcinoma (SCC), and 50 cases of unspecified subtype (Table 2). As most primary carcinomas of the stomach are adenocarcinomas, gastric carcinomas were stratified according to subsite (rather than histological subtype). The sample of gastric cases comprised 25 cases from each subsite of the stomach, and 50 cases of unspecified subsite (Table 2). Data on 98 cases of gastric carcinoma classified as SCCs were also checked opportunistically, as this number of cases appeared suspiciously high.

Opportunistic validity checks were also performed on abstracted hospital case notes of patients recorded as ‘alive’

*Received 30 March 1998*
*Revised 19 November 1998*
*Accepted 24 November 1998*

*Correspondence to:* R Sutton

**Table 1**

| | Subtype | | Subsite | |
|---|---|---|---|---|
| | ICD-O | This study | ICD-O | This study |
| Oesophagus | Adenocarcinoma | Adenocarcinoma | Upper third | Upper third |
| | Squamous cell | Squamous cell | Cervical | Upper third |
| | NOS | Unspecified | Middle third | Middle third |
| | Undifferentiated | Others | Abdominal | Lower third |
| | Adenosquamous | Others | Lower third | Lower third |
| | Small cell | Others | Overlapping | Overlapping |
| | | | [a]Thoracic | Unspecified |
| | | | NOS | Unspecified |
| Stomach | Adenocarcinoma | Adenocarcinoma | Cardia | Cardia |
| | NOS | Unspecified | Fundus | Fundus |
| | Undifferentiated | Other | Body | Subfundus |
| | Squamous cell | Others | Greater | Subfundus |
| | Small cell | Others | Lesser curve | Subfundus |
| | Carcinoid | Others | [a]Anterior | Subfundus |
| | | | [a]Posterior | Subfundus |
| | | | Antrum | Subfundus |
| | | | Pylorus | Subfundus |
| | | | NOS | Subfundus |

NOS, not otherwise specified. [a]We described these as unspecified as the numbers were few (nine in the whole study), and it is unclear how many upper third and lower third carcinomas might have been included in this subsite category.

**Table 2** Data verification of 100 oesophageal carcinomas and 200 gastric carcinomas

| | Oesophagus | | | | Stomach[a] | | | | | | | |
|---|---|---|---|---|---|---|---|---|---|---|---|---|
| | Adeno[b] | SCC[c] | Unspec[d] | Total | Cardia | Fundus | Body | Greater curve | Lesser curve | Overlapping | Unspec[d] | Total |
| No. checked | 25 | 25 | 50 | 100 | 25 | 25 | 25 | 25 | 25 | 25 | 50 | 200 |
| No. correct | 24 | 25 | 47 | 95 | 19 | 22 | 23 | 24 | 23 | 18 | 42 | 171 |
| Subtype error | 0 | 0 | 3 | 3 | 0 | 0 | 0 | 0 | 0 | 0 | 0 | 0 |
| Subsite error | 1 | 0 | 1 | 2 | 6 | 3 | 2 | 1 | 2 | 7 | 8 | 29 |

[a] Using original classification (ICD-O, Percy et al, 1990) held by MCCR; [b]adenocarcinoma; [c]squamous cell carcinoma; [d]unspecified.

10 years after the diagnosis of carcinoma, comprising 76 cases (1.4%) of oesophageal carcinoma and 142 cases (1.3%) of gastric carcinoma.

### Data analysis

The annual subtype-specific and subsite-specific incidences of oesophageal and gastric carcinomas from 1974 to 1993 were calculated as cumulative rates (0–79 years). The cumulative rate was calculated as the sum of the age-specific incidence rates for each year from birth to 79 years, and estimates the cumulative risk of developing the disease before the age of 79 years.

All histologically proven cases of adenocarcinoma of the oesophagus and stomach in which the subsite was also specified were extracted from the data, and subdivided into four groups according to subsite: upper two-thirds of the oesophagus, lower one-third of the oesophagus, cardia and subcardia (fundus and subfundus combined) of the stomach. A comparison of all identifiable epidemiological and clinical features of adenocarcinoma at these four subsites was then performed.

### Statistical analysis

Linear regression was used to study the changes in incidence over time. Comparisons between different subtypes and between different subsites were performed using $\chi^2$ tests, the exception being age at diagnosis, which was compared using Mann–Whitney *U*-test. The Kaplan–Meier method was used to determine survival, and log-rank tests to compare survival at different subsites.

### RESULTS

### Identification of cases

There were 5322 cases of primary oesophageal carcinoma and 10 535 cases of primary gastric carcinoma registered between 1974 and 1993 (Tables 3 and 4). Histological proof of diagnosis was obtained in 69% of oesophageal cases and 56% of gastric cases, although in the last quinquennia studied (1989–1993), this had risen to 72% and 71% respectively.

© Cancer Research Campaign 1999

**Table 3** Sex, age and subsite details on the histological subtypes of all cases of oesophageal carcinoma from 1974 to 1993 inclusive

| | | Adenocarcinoma (%) | Squamous (%) | Unspecified (%) | Others (%) | Total (%) |
|---|---|---|---|---|---|---|
| Total cases | | 1532 (29) | 1922 (36) | 1689 (32) | 179 (3.4) | 5322 |
| Sex | Male | 1160 (76) | 817 (42) | 899 (53) | 102 (57) | 2978 (56) |
| | Female | 372 (24) | 1105 (58) | 790 (47) | 77 (43) | 2344 (44) |
| Age (years) | Median | 68 | 69 | 75 | 68 | 70 |
| | Range | 25–95 | 32–101 | 34–100 | 43–94 | 25–101 |
| Subsite | Upper | 31 (2) | 210 (11) | 79 (5) | 11 (6) | 331 (6) |
| | Middle | 122 (8) | 540 (28) | 208 (12) | 39 (22) | 909 (17) |
| | Lower | 1023 (67) | 688 (36) | 474 (28) | 81 (45) | 2266 (43) |
| | Overlapping | 34 (2) | 44 (2) | 26 (2) | 6 (3) | 110 (2) |
| | Unspecified | 322 (21) | 440 (23) | 902 (54) | 42 (24) | 1706 (32) |
| Not treated | | 555 (36) | 660 (34) | 1156 (68) | 83 (46) | 2459 (46) |
| too advanced | | 412 (74) | 450 (68) | 558 (48) | 63 (76) | 1483 (60) |
| unfit | | 95 (17) | 131 (20) | 259 (23) | 15 (18) | 500 (20) |
| not known | | 48 (9) | 79 (12) | 339 (29) | 5 (6) | 471 (19) |
| DC registered[a] | | 203 (13) | 231 (12) | 767 (45) | 27 (15) | 1226 (23) |

[a] Cases registered from death certificates; MCCR tracks death certificates back to complete records in two-thirds of cases (Seddon and Williams, 1997)

**Table 4** Sex, age and subtype details on the subsites of all cases of gastric carcinoma from 1974 to 1993 inclusive

| | | Cardia (%) | Fundus (%) | [a]*P*-value | Subfundus (%) | [b]*P*-value | [c]Unspec (%) | Total (%) |
|---|---|---|---|---|---|---|---|---|
| Total cases | | 1508 (14) | 386 (4) | | 2816 (27) | | 5825 (55) | 10535 |
| Sex | Male | 1090 (72) | 245 (64) | 0.004 | 1760 (63) | < 0.00001 | 3182 (55) | 6267 (60) |
| | Female | 418 (28) | 141 (36) | | 1056 (37) | | 2653 (45) | 4268 (40) |
| Age (years) | Median | 69.0 | 70.0 | 0.0006 | 71.0 | < 0.00001 | 73 | 72 |
| | Range | 24–101 | 24–95 | | 19–101 | | 20–103 | 19–103 |
| Subtype | [d]Adenoca | 1130 (75) | 195 (51) | < 0.0001 | 1871 (66) | < 0.00001 | 2128 (37) | 5324 (50) |
| | [c]Unspec | 333 (22) | 179 (46) | | 822 (29) | | 3598 (62) | 4932 (47) |
| | Others | 45 (3) | 12 (3) | | 123 (4) | | 99 (2) | 279 (3) |

[a]*P*-value represents statistical comparison of carcinoma of the cardia and of the fundus, using $\mathrm{X}^2$ and Mann–Whitney *U*-tests; [b]*P*-value represents statistical comparison of carcinoma of the cardia and of the subfundus; [c]unspecified; [d]adenocarcinoma.

### Data verification

Data verification revealed that three of 100 oesophageal carcinomas had been mis-classified by subtype (all three had been classified as unspecified, but were adenocarcinomas), and that two of 100 had been mis-classified by subsite (both were carcinomas of the cardia, Table 2). There were no errors of subtype classification in 200 cases of gastric carcinoma examined. However, 29 cases (15%) contained errors of subsite classification, mostly occurring in tumours originally classified as cardia, overlapping or unspecified subsites. On review of pathology reports and hospital records, the subsite of origin was identified in eight of 50 (16%) cases originally classified as unspecified, with one case originating in the cardia, and seven cases in the subcardia. Of 25 cases originally classified as cardia, five (20%) were actually carcinomas of the lower oesophagus; the mis-classification of lower oesophageal tumours as carcinomas of the cardia was the most common error detected by data verification.

Of 98 cases originally classified as SCCs of the stomach, pathology reports were available for 85 cases (87%): 66 cases (67%) were SCC arising in the oesophagus, four (4%) cases were adenocarcinomas of the stomach and four (4%) did not have a histological diagnosis (unspecified). All of these cases were recoded correctly. A further two cases originated outside the upper gastrointestinal tract (larynx and anus) and were excluded from the analysis, leaving nine cases (9%) classified as SCC of the stomach. The remaining 13 cases (13%) in which pathology reports were not available remained classified as SCCs of the stomach.

Validity checks on abstracted hospital case notes of patients alive 10 years after the diagnosis of carcinoma revealed that nine of 76 cases (12%) of oesophageal carcinoma, and 23 of 142 cases (16%) of gastric carcinoma, had died. These were recoded correctly.

### Histological and subsite classification

Over the 20-year study period, there were more SCC (1922) than adenocarcinomas (1532) of the oesophagus (Table 3). The ‘other’ subgroup consisted predominantly of undifferentiated (96), small cell (47) and adenosquamous (18) carcinomas. About one-third (1689) of oesophageal carcinomas were classified as unspecified subtype, although data verification suggests that subtype was known in 6% of a random sample of these cases. Almost one-half (767) of carcinomas of unknown subtype were registered by death certificate; with routine tracking back, MCCR usually gains complete information in two-thirds of such cases (Seddon and Williams, 1997). Overall, oesophageal carcinomas of unspecified subtype had a similar male to female ratio, and a similar upper to middle to lower subsite ratio as oesophageal carcinomas in total (Table 3), and so were also likely to contain a similar subtype ratio. Patients with carcinomas of unspecified subtype were older ($P < 0.0001$, Mann–Whitney *U*-test), and less likely to have

 © Cancer Research Campaign 1999

received treatment ($P < 0.0001$, $\chi^2$ test) than other oesophageal carcinomas (Table 3). Hence, it is reasonable to assume that patients with carcinomas of unspecified subtype were representative of the dataset as a whole with regards to subtype and subsite, although they were older and probably had more advanced disease. Adenocarcinomas and SCCs had significantly different male to female ratios at 3:1 (1160:372) and 2:3 (817:1105) respectively ($P < 0.0001$, $\chi^2$ test), and significantly different upper: middle:lower third subsite ratios at 1:4:33 (31:122:1023) and 1:3:3 (210:540:688) respectively ($P < 0.00001$, $\chi^2$ test). Notably, 87% (1023 cases) of oesophageal adenocarcinomas in which the subsite was specified occurred in the lower third of the oesophagus.

In the stomach, adenocarcinoma represented 94% (5324) of all histological diagnoses of gastric carcinoma (Table 4). The remaining histologically verified gastric carcinomas, classified as ‘other’ (259), were almost entirely undifferentiated carcinomas (231). Sixty per cent (2965) of gastric carcinomas without a histological diagnosis were registered by death certificate, making further details difficult to acquire, although it is likely that almost all of these carcinomas were adenocarcinomas. Regarding subsite classification, 14% (1508) of gastric carcinomas originated in the cardia, 4% (386) in the fundus and 27% (2816) in the subfundus, with 55% (5825) being classified as subsite unspecified (Table 4). Almost half (2737) of these cases classified as subsite unspecified were registered by death certificate, again limiting the information available. Interestingly, the subtype was known in 39% (2227) of cases in which the subsite was unspecified. Nevertheless, gastric carcinomas of unspecified subsite were commoner in older patients (vs gastric carcinomas with specified subsite, $P < 0.001$, Mann–Whitney *U*-test), and commoner in women (vs gastric carcinomas with specified subsite, $P < 0.001$, $\chi^2$ test), as were carcinomas arising in the subfundic regions of the stomach (vs carcinoma of the cardia, $P < 0.0001$, $\chi^2$ test, Table 4). For calculation of subsite-specific incidences, gastric carcinomas of unspecified subsite were included in the subfundus group, as previous studies suggest that the majority of gastric carcinomas occur in the subfundus (Antonioli et al, 1982; Correa, 1994; Locke et al, 1995), and validity checks on 50 cases of gastric carcinoma of unspecified subsite revealed mis-classification errors in eight cases (16%), with seven of these eight cases (88%) actually arising in the subfundus. Also, classification of all gastric carcinomas with unspecified subsite as subfundus carcinomas prevents over-estimation of any rise in the incidence of carcinoma of the cardia.

### Temporal trends in incidence

From 1974 to 1993, the cumulative incidence (0–79 years) of oesophageal carcinoma increased significantly in males from 1.25 to 1.72% ($P = 0.008$, linear regression), and remained relatively constant in females at 0.7%.

The increase in oesophageal carcinoma from 1974 to 1993 was due to a rapid rise in adenocarcinoma, the cumulative incidence (0–79 years) of which increased from 0.32 to 0.85% in males ($P < 0.00001$, linear regression), and from 0.07 to 0.14% in females ($P < 0.0001$, linear regression, Figure 1). The cumulative incidence (0–79 years) of SCC of the oesophagus remained relatively constant in males at 0.43%, and increased significantly in females from 0.37 to 0.39% ($P = 0.004$, linear regression, Figure 1). In the last 5 years of the study, the male:female ratios for adenocarcinoma and SCC of the oesophagus were 5:1 and 1:1 respectively. There was a small but significant decrease in carcinomas classified as subtype unspecified in both males ($P = 0.02$, linear regression) and females ($P = 0.003$, linear regression, Figure 1). The annual cumulative rate of oesophageal carcinoma classified as ‘others’ did not change significantly between 1974 and 1993, and was too small to be represented on the graph. Analysis of the subsite-specific incidence of oesophageal carcinoma was performed for cases registered between 1974 and 1992, as a number of cases registered in 1993 had incomplete details on subsite classification. There were significant increases in the incidence of carcinomas of the upper, middle and lower thirds of the oesophagus in both males and females. The largest increases occurred in carcinomas of the lower third, the cumulative incidence (0–79 years) of which increased from 0.53 to 0.90% in males ($P < 0.0001$, linear regression), and from 0.20 to 0.27% in females ($P = 0.0002$, linear regression, Figure 2); these increases reflect the rise in incidence of adenocarcinoma, 87% of which occurred in the lower oesophagus.

There was a steady, significant decline in the cumulative incidence (0–79 years) of gastric carcinoma between 1974 and

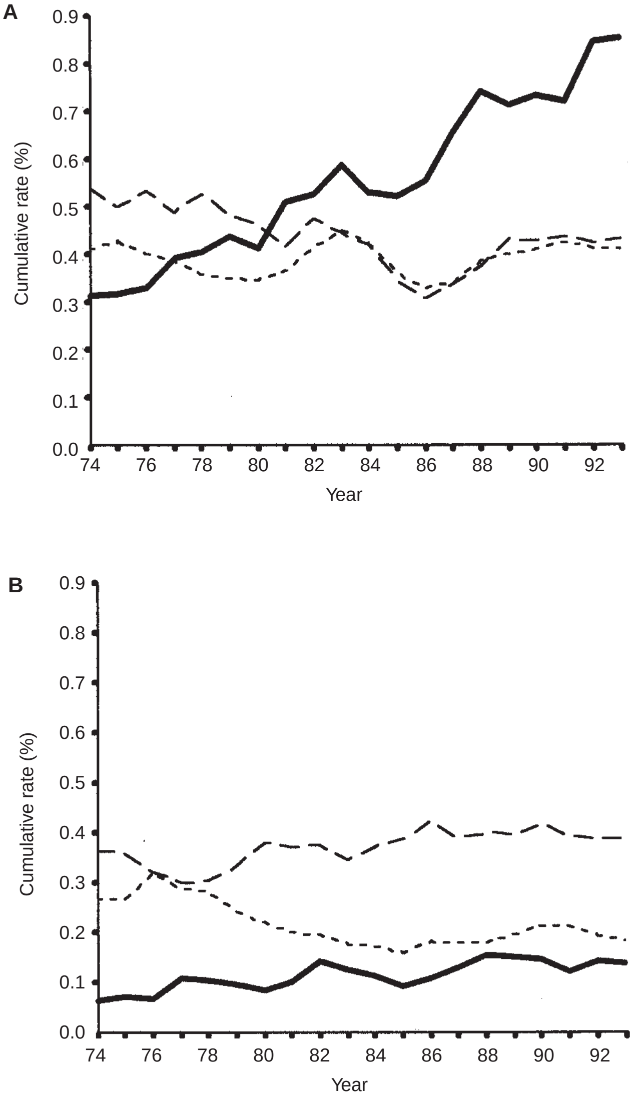

**Figure 1** Subtype-specific cumulative rates (%) of oesophageal carcinoma (3-year rolling averages) in males (**A**) and females (**B**) from 1974 to 1993 inclusive. (——) Adenocarcinoma; (– –) unspecified; (– – –) squamous

© Cancer Research Campaign 1999

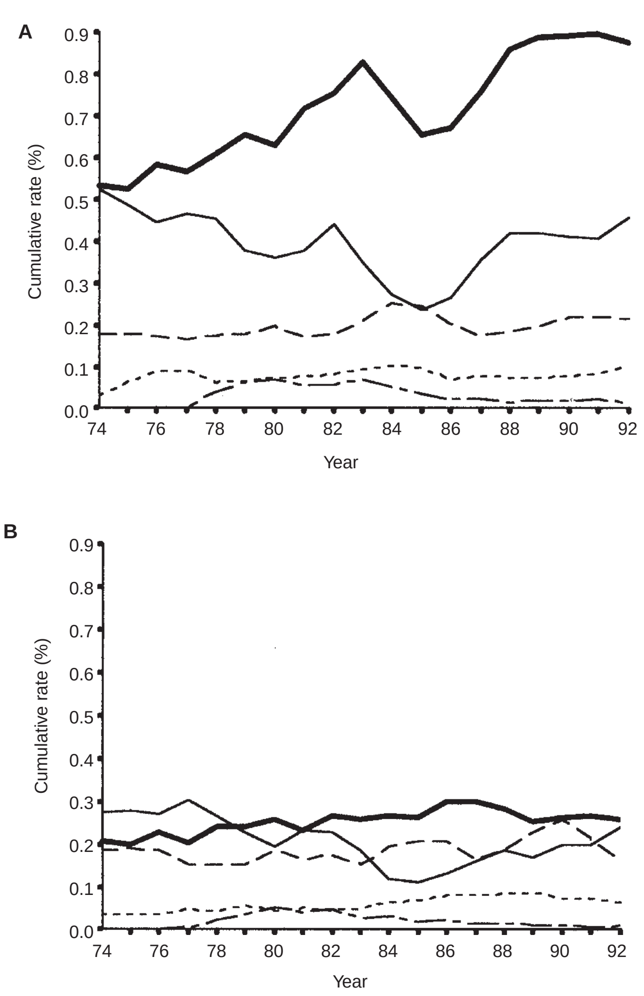

**Figure 2** Subsite-specific cumulative rates (%) of oesophageal carcinoma (3-year rolling averages) in males (top) and females (bottom) from 1974 to 1992 inclusive. (– – –) Upper one-third; (— –) middle one-third; (——) lower one-third; (— —) overlapping; (——) unspecified

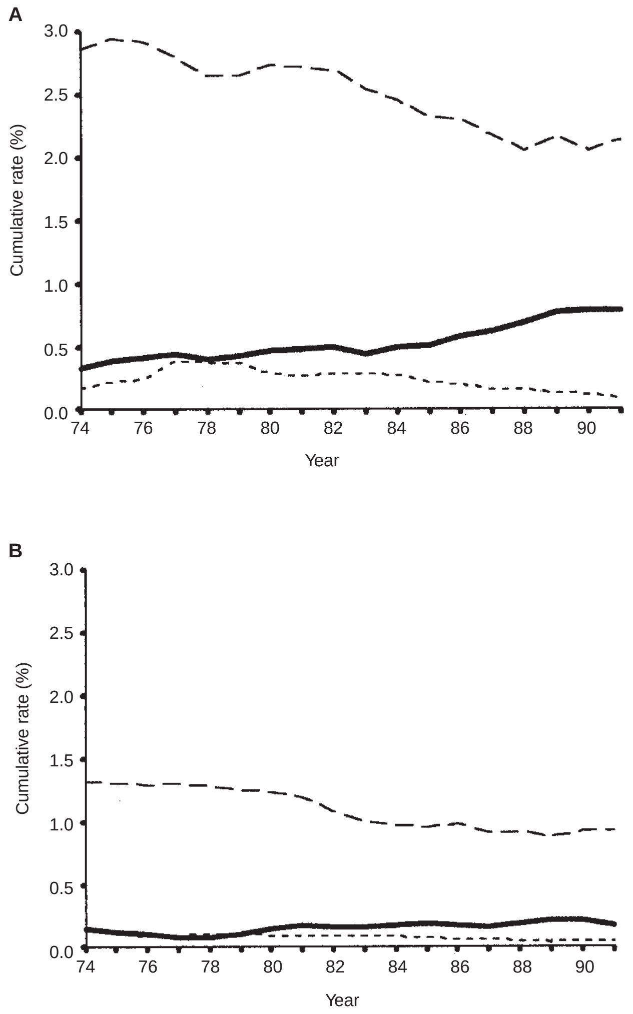

**Figure 3** Subsite-specific cumulative rates (%) of gastric carcinoma (3-year rolling averages) in males (top) and females (bottom) from 1974 to 1991 inclusive. (——) Cardia; (– – –) fundus; (— –) subfundus

1993 in males (3.5 to 2.8%) ($P$ < 0.0001, linear regression), and in females (1.5 to 1.0%) ($P$ < 0.0001, linear regression). As 94% of all gastric carcinomas diagnosed by histological means were adenocarcinomas, analysis of the subtype-specific incidence rates was not performed. The subsite-specific incidence of gastric carcinoma for males and females are displayed for 1974 to 1991 only (Figure 3), as a number of cases registered in 1992 and 1993 had incomplete subsite details. The only subsite of the stomach which did not decline in incidence over the study period was the cardia, the cumulative incidence (0–79 years) of which increased from 0.39 to 0.79% in males ($P$ < 0.0001, linear regression), and from 0.12 to 0.21% in females ($P$ = 0.0001, linear regression, Figure 3). Towards the end of the study, the male:female ratio for cardia and for subcardia tumours were 5:1 and 2:1 respectively.

### Subsite comparison of adenocarcinoma

The epidemiological and clinical comparison of adenocarcinoma at four different subsites (upper two-thirds of the oesophagus, lower third of the oesophagus, cardia and subcardia) was performed using only data in which both the subtype and subsite were specified (Table 5). The majority of the subsite data for the oesophagus were derived from ICD-O classification as upper (312), middle (909) or lower third (2232), and not cervical (19), thoracic (nine, all placed within unspecified category because of uncertain overlap) or abdominal (34), the alternative form of this classification. Adenocarcinomas of the fundus and of the subfundus had similar changes in incidence and were similar for age at diagnosis and sex ratio (Table 4), and so were grouped together as adenocarcinomas of the subcardia. Adenocarcinomas of the lower oesophagus and of the cardia had a significantly greater male predominance than adenocarcinomas elsewhere in the upper gastrointestinal tract ($P$ < 0.001, $\chi^2$ test), and both occurred at a younger age ($P$ < 0.0001, Mann–Whitney $U$-test, Table 5). As described above, the incidence of adenocarcinoma of the lower oesophagus and of the gastric cardia were similar throughout the 20-year study period, both increasing to a similar degree, whilst carcinomas of the subcardia declined. An almost identical percentage of patients with adenocarcinoma of the oesophagus and of the cardia were smokers, and this percentage was significantly

© Cancer Research Campaign 1999

**Table 5** Comparison of epidemiological features of adenocarcinoma at different subsites of the upper gastrointestinal tract from the MCCR dataset 1974 to 1993 inclusive. The upper part of the Table summarizes the data, whereas the lower part provides statistical comparison of the four subsite groups

| | | Oesophagus | | Stomach | |
|---|---|---|---|---|---|
| | | **Upper two-thirds** | **Lower one-third** | **Cardia** | **Subcardia** |
| Total cases | | 153 | 1023 | 1130 | 2066 |
| Sex | Males | 99 | 814 | 848 | 1347 |
| | Females | 54 | 209 | 282 | 719 |
| | M:F ratio | 1.8 : 1 | 4 : 1 | 3 : 1 | 1.9 : 1 |
| Age (years) | Median | 69.0 | 67.0 | 68.0 | 70.0 |
| | Range | 39–92 | 25–95 | 24–97 | 19–101 |
| Incidence | Males | Increase × 1.2 | Increase × 3 | Increase × 2.5 | Decrease |
| | Females | Increase × 1.2 | Increase × 2 | Increase × 1.8 | Decrease |
| Smoking | Yes | 54 | 319 | 365 | 685 |
| | No | 66 | 384 | 444 | 1062 |
| | Smokers (%) | 45.0 | 45.4 | 45.0 | 39.2 |
| | Upper vs lower | Lower vs cardia | Lower vs subcardia | Cardia vs subcardia | |
| Age[a] | NS[c] | NS[c] | < 0.0001 | < 0.0001 | |
| Sex[b] | < 0.0001 | NS[c] | < 0.0001 | < 0.00001 | |
| Smoking[b] | NS[c] | NS[c] | < 0.0001 | < 0.00001 | |

[a] Mann–Whitney *U*-test; [b]$\chi^2$ test; [c]$P > 0.05$.

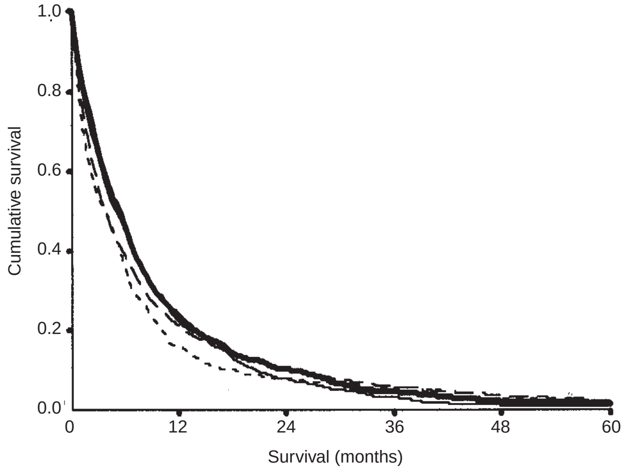

| Subsite | Upper two-thirds | Lower one-third | Cardia |
|---|---|---|---|
| Lower one-third | 0.12 | – | – |
| Cardia | 0.01 | 0.06 | – |
| Subcardia | 0.0006 | <0.0001 | 0.03 |

**Figure 4** Survival of patients with adenocarcinoma of the upper two-thirds and lower one-third of the oesophagus, cardia and subcardia of the stomach from 1974 to 1993 inclusive. Tabulated *P*-values are from comparison of survival at different subsites (log-rank tests). (— –) Subcardia; (——) cardia; (— —) lower one-third; (– – –) upper two-thirds greater than those patients with adenocarcinoma of the subcardia ($P < 0.0001$, $\chi^2$ test). Patients with adenocarcinoma of the lower oesophagus and of the cardia had similar survival ($P = 0.06$, log-rank), and both had significantly worse survival than patients with adenocarcinoma of the subcardia ($P = 0.03$, log-rank, Figure 4). Overall, survival from adenocarcinomas of the oesophagus and stomach was universally poor at all subsites, with 34%, 31%, 29% and 21% of patients with adenocarcinomas of the subcardia, cardia, lower one-third and upper two-thirds of the oesophagus, respectively, surviving 12 months following diagnosis.

### DISCUSSION

The rise in incidence of oesophageal carcinoma in Merseyside and Cheshire from 1974 to 1993 was almost entirely due to an increase in adenocarcinoma of the lower third, the incidence of which trebled in males and doubled in females. There was a decrease in oesophageal carcinoma in 1985, occurring against a background of overall increase, and this is likely to reflect data processing artefact when MCCR was undergoing recomputerization. The rise in oesophageal adenocarcinoma in males is four times greater than the decrease in carcinomas with unspecified subtype, and so cannot be explained by increasing diagnostic precision alone. Carcinomas of unspecified subtype had similar sex and subsite distributions to oesophageal carcinomas as a whole, and it is reasonable to assume that the subtype distribution was similar. Therefore, changes in the incidence of carcinomas of unspecified subtype is unlikely to have significantly altered the pattern of changes in subtype-specific incidence. In fact, three errors of subtype classification identified by validity checks on 100 cases of oesophageal carcinomas all occurred in adenocarcinomas misclassified as unspecified subtype. Subtype mis-classification error in this study may have masked an even greater rise in adenocarcinoma of the oesophagus. Increases in the incidence of oesophageal adenocarcinoma have also been reported in North America (Blot et al, 1991), Scotland (McKinney et al, 1995) and Norway (Hansen et al, 1997). Similarly, the increase in incidence of carcinomas of the lower third of the oesophagus cannot be fully accounted for by the much smaller decrease in incidence of carcinomas classified as unspecified subsite. The rise in adenocarcinoma of the lower oesophagus may be underestimated since data verification revealed that five of 25 cases originally classified as cardia had actually arisen from the lower oesophagus. These subsite errors in gastric carcinoma, if representative of all registered cases, may have caused an overestimation (20% of 1508 = 301 cases) of the increase in carcinoma of the cardia, which doubled in males and females during the study period. Conversely, the rise in carcinoma of the cardia may have been underestimated by the categorization of gastric carcinomas of unspecified subsite

© Cancer Research Campaign 1999

as carcinomas of the subcardia, as this group may have included a small number of carcinomas of the cardia. Data verification revealed that the subsite was known in eight of 50 gastric carcinomas classified as unspecified subsite, with one of eight (12%) arising in the cardia. Carcinomas of the cardia account for between 0 and 27% of all gastric carcinomas, depending on the years studied (Antonioli et al, 1982; Correa, 1994; Locke et al, 1995). If at least 4% (233 cases) of gastric carcinomas of unspecified subsite in this study were carcinomas of the cardia, as suggested by data verification and other studies, then this underestimation would balance the overestimation caused by the mis-classification of lower oesophageal carcinomas as carcinomas of the cardia. Thus the increase in incidence of carcinoma of the cardia is likely to be real. The incidence of carcinoma at all other sites of the stomach other than the cardia declined during the same time period, including those originating from the fundus. Other studies have identified decreases in the incidence of distal gastric carcinomas, but have failed to demonstrate changes in incidence of proximal (cardia and fundus combined) gastric carcinomas (Locke et al, 1995; Hansen et al, 1997). The stable incidence rates of proximal carcinomas in these studies may have hidden increases in carcinomas of the cardia and decreases in carcinomas of the fundus, both of which were evident in our study.

Although the effects of mis-classification can never be completely overcome in a population-based study, adenocarcinomas of the lower oesophagus and of the cardia were strikingly similar in all the clinical and epidemiological features studied, and both differed significantly from carcinoma of the subcardia, a difference that has been previously noted in a non-population based study of 166 patients (Wang et al, 1986). In order to minimize any classification errors, only adenocarcinomas with a specified subsite were included in this part of the study. Patients with adenocarcinoma of the cardia had similar survival to patients with adenocarcinoma of the lower oesophagus, and both had a significantly worse survival than patients with adenocarcinoma of the subcardia. Data describing the TNM (tumour, node, metastasis) classification of carcinomas, a method of assessing stage that uses the subsite and subtype classification of ICD-O (Spiessl et al, 1992; Sobin and Wittekind, 1997), were not recorded in this population-based study, and so we cannot comment on whether a more favourable stage distribution might account for the better survival in adenocarcinoma of the subcardia. SEER data from the USA have also shown that carcinomas of the subcardia have a better prognosis than carcinomas of the cardia (Thomas and Sobin, 1995). However, survival from adenocarcinoma was poor at all sites of the oesophagus and stomach. In our study, errors relating to survival data were similar for both oesophageal and gastric carcinomas, mainly affecting a minority of long-term survivors, and therefore not noticeably affecting overall survival. As discussed, subsite classification errors mainly involved adenocarcinomas of the lower oesophagus being registered as adenocarcinomas of the cardia. The inclusion of lower oesophageal adenocarcinomas in the cardia subgroup could contribute to, but is unlikely to explain all, the similarities between adenocarcinomas at these subsites; this inclusion also highlights the clinical and pathological difficulties in distinguishing between them.

The aetiology of adenocarcinoma of both the lower oesophagus and of the cardia is likely to be associated with gastro-oesophageal reflux disease and Barrett's oesophagus (Cameron et al, 1995; Chow et al, 1995), in particular chronic duodenogastro-oesophageal reflux resulting in intestinal metaplasia (Caldwell et al, 1995; Miwa et al, 1996). The rise in incidence of adenocarcinoma around the gastro-oesophageal junction (GOJ) may reflect an increased prevalence of gastro-oesophageal reflux disease (Loof et al, 1993). Adenocarcinomas of the lower oesophagus and cardia are not related to *Helicobacter pylori* infection (Ricaurte et al, 1996), which is significantly more prevalent in patients with carcinoma of the subcardia (Hansson et al, 1995).

Adenocarcinomas of the lower oesophagus and cardia are also very similar in their growth pattern, degree of differentiation and presence of nodal metastases at resection, and, not surprisingly, the most common complaint in both oesophageal and cardia carcinomas is dysphagia, which is present in over 75% of cases (Kalish et al, 1984). Thus in addition to aetiology, the epidemiology, clinical features, pathology and survival of adenocarcinomas of the lower oesophagus and cardia have been shown to be similar in small studies of selected patients, but our study is the first to confirm these last four similarities in population-based data. Not only did we find carcinoma of the cardia to resemble lower oesophageal carcinoma in all these features, but both were significantly different from carcinoma of the subcardia.

The remarkable similarity between adenocarcinomas of the lower oesophagus and of the cardia suggests that the arbitrary division of these carcinomas into oesophageal and gastric carcinomas is inappropriate. Previous pathological subsite classifications based on resected specimens have involved determination of the centre of the carcinoma and measurement of the distance to the GOJ (Holscher et al, 1995), or have required estimation of where the majority of the carcinoma is situated (Tachimori et al, 1996). Indeed, the identification of the origin of a tumour around the GOJ can be even more difficult preoperatively, especially in the presence of a hiatus hernia and/or Barrett's oesophagus, where the site of the GOJ and cardia may be difficult to determine endoscopically or radiologically (Spechler and Goyal, 1986; Csendes et al, 1993). In the presence of chronic inflammatory changes resulting from gastro-oesophageal reflux disease, it may even be difficult to determine the precise location of the cardia by histological means (Riddell, 1996). It is therefore not surprising that, in our study, data verification identified a number of cases of subsite mis-classification involving carcinoma of the lower oesophagus and gastric cardia. Indeed, subsite mis-classification of carcinomas around the GOJ would appear inevitable within the present ICD-O classification scheme, which does not code carcinoma of the GOJ as a distinct entity, and which also includes overlapping subsites for carcinomas of the stomach. Furthermore, ICD-O contains two alternative subsite classifications for oesophageal carcinoma (cervical, thoracic and abdominal versus upper, middle and lower) within ICD-O, adding further confusion. This study shows how infrequently the first of these two alternatives is used. Yet the ICD-O scheme for the oesophagus and stomach is identical to that of ICD 9 and ICD 10 on which it is based (Percy et al, 1990), is the international standard, is preferred by the International Agency for Research on Cancer of the World Health Organization, and is the most widely used within cancer registries worldwide (Whelan and Young, 1997). In addition, the most recent version of ICD-O (Percy et al, 1990) failed to address the difficulties of classification discussed above.

In view of these difficulties, and of the recent evidence of similarities between carcinomas of the lower oesophagus and cardia, a new subsite classification of carcinomas of the upper gastrointestinal tract from the pharynx to the duodenum is needed. An alternative classification for carcinomas around the GOJ has

 *© Cancer Research Campaign 1999*

**Table 6**

| Site | Subsite | Definition |
|---|---|---|
| Oesophagus | Upper third | Cricopharyngeal sphincter to thoracic inlet |
| | Middle third | Thoracic inlet to 8th thoracic vertebra |
| | Lower third | Below 8th thoracic vertebra and above GOJ |
| GOJ | GOJ | Involves GOJ, either from above or below |
| Stomach | Proximal | Proximal to incisura angularis |
| | Distal | Distal to incisura angularis |
| | Overlapping | Involves more than 50% of each subsite |

recently been described in which carcinomas are subdivided into lower oesophageal, GOJ and cardia depending on the distance from the centre of the carcinoma to the GOJ (Siewert and Stein, 1996, 1998). Unfortunately, as the distances to the GOJ may be small and the carcinomas may be large, this classification is difficult to implement when the diagnosis is made by endoscopy only and the patient does not undergo surgical resection, which is the situation in the majority of cases (Stipa et al, 1992). Furthermore, our study has shown that the use of cardia as a subsite for carcinomas around the GOJ is prone to error, principally due to difficulties in determining its precise location, as discussed above. Our study also suggests that difficulties exist with implementation of the ICD-O subsite classification for carcinomas of the stomach, as 39% of gastric carcinomas classified as subsite unknown actually had a histological diagnosis, indicating that, despite an endoscopy, the subsite could still not be accurately identified in a number of cases. Hence, we propose a simpler pragmatic ‘Liverpool’ classification that can be applied to tumours diagnosed endoscopically, and takes into account these new findings. Carcinomas of the upper gastrointestinal tract between the pharynx and the duodenum should be initially classified into carcinomas of the oesophagus, GOJ and stomach, as shown in Table 6. Oesophageal carcinomas occur below the cricopharyngeal sphincter and above the GOJ, and should be subdivided into upper, middle and lower thirds. When upper tumours become middle, and middle tumours become lower, is and will be determined grossly at endoscopy in the majority of patients, for which detail is given in Table 6 as a guide. Any carcinoma that encroaches upon the GOJ from above or from below should be classified as GOJ, and will therefore include some of the carcinomas previously classified as lower oesophageal and the majority of carcinomas previously classified as cardia. Further subsite division of carcinomas of the GOJ, as proposed by Siewert and Stein (1996, 1998) would be impossible to implement by endoscopy, and may also be difficult to implement for those patients undergoing resection of large carcinomas. Carcinomas below the GOJ that do not involve the GOJ should be classified as gastric, which should be subdivided into proximal, distal and overlapping subsites. Carcinomas of the fundus were similar both clinically and epidemiologically to carcinomas of the subfundus, the majority of which in our study were carcinomas of the body of the stomach; carcinomas of the fundus and of the body of the stomach are therefore grouped together as proximal carcinomas. The difficulties encountered with the ICD-O subsite classification prevented detailed analysis of carcinomas at different subsites of the stomach, particularly carcinomas of the pylorus and antrum, where again there exists a degree of overlap. The ‘Liverpool’ system simply classifies all carcinomas distal to the incisura angularis, which is easily identified on endoscopy, as distal carcinomas. Distal carcinomas would therefore replace carcinomas of the pylorus and antrum, and implementation of this classification would permit future clinical and epidemiological comparisons of proximal and distal carcinomas, which is difficult under the present ICD-O system. Overall, use of the ‘Liverpool’ classification would make registration of carcinoma of the oesophagus and stomach simpler and more accurate, thereby clarifying the epidemiological surveillance of upper gastrointestinal carcinomas, and is in step with changes in diagnostic techniques.

Accurate histological and subsite classification of upper gastrointestinal carcinomas is important in determining treatment. The extent of surgical resection for carcinoma around the GOJ remains to be determined (Stipa et al, 1996), as does the role of chemoradiotherapy (Hennessy, 1996). Part of the difficulty in determining appropriate treatment for carcinoma around the GOJ stems from problems in comparing results from different series, in turn partly the result of the lack of an accurate subsite classification system (Stipa et al, 1992). The widespread application of our proposed subsite classification to carcinoma of the oesophagus and stomach would aid the planning and interpretation of future treatment trials for carcinoma of the GOJ.

In summary, this study confirms a simultaneous, dramatic increase in the incidence of adenocarcinoma of the lower oesophagus and of the gastric cardia in the last 20 years. Despite the confusion created by the present ICD-O classification of carcinoma at the GOJ, it is apparent that adenocarcinoma of the lower oesophagus and of the cardia are similar in aetiology, epidemiology and clinico-pathological features; it is compelling evidence that they are the same disease. This is taken into account in a new subsite classification of carcinoma of the oesophagus and of the stomach, which includes a category for carcinoma of the GOJ. Use of this new subsite classification is likely to help clarify the changing epidemiology of carcinomas of the oesophagus and stomach, and to allow comparison of different treatment strategies for these carcinomas.

### ACKNOWLEDGEMENTS

K Dolan was supported in a Surgical Research Fellowship by the Ursula Keyes Trust.

### REFERENCES

Antonioli DA and Goldman H (1982) Changes in the location and type of gastric adenocarcinoma. *Cancer* **50**: 775–781

Blot WJ, Devesa SS, Kneller RW and Fraumeni JF (1991) Rising incidence of adenocarcinoma of the oesophagus and gastric cardia. *JAMA* **265**: 1287–1289

Caldwell MT, Lawlor P, Byrne JP, Healy M, O’Moore RR and Hennessy TP (1995) Ambulatory oesophageal bile reflux monitoring in Barrett’s oesophagus. *Br J Surg* **75**: 657–660

Cameron AJ, Lomboy CT, Pera M and Carpenter HA (1995) Adenocarcinoma of the oesophagogastric junction and Barrett’s oesophagus. *Gastroenterology* **109**: 1541–1546

Chow WH, Finkle WD, McLaughlin JK, Frankl H, Ziel HK and Fraumeni JF (1995) The relation of gastroesophageal disease and its treatment to adenocarcinomas of the oesophagus and gastric cardia. *JAMA* **274**: 474–477

Correa P and Chen VW (1994) Trends in cancer incidence and mortality. *Cancer Surv* **19/20**: 55–76

Craanen ME, Deker W, Blok D, Ferwerda J and Tytgat GNJ (1992) Time trends in gastric cancer; changing patterns of type and location. *Am J Gastroenterology* **87**: 572–579

Csendes A, Maluenda F, Braghetto I, Csendes P, Henriquez A and Queseda M (1993) Location of the lower oesophageal sphincter and the squamo-columnar mucosal junction in 109 healthy controls and 778 patients with different degrees of endoscopic oesophagitis. *Gut* **34**: 21–27

© Cancer Research Campaign 1999

Hansen S, Wiig JN, Giercksky KE and Tretli S (1997) Esophageal and gastric carcinoma in Norway 1958–1992: incidence time trend variability according to morphological subtypes and organ subsites. *Int J Cancer* **71**: 340–344

Hansson LE, Engstrand L, Nyren O and Lindgren A (1995) Prevalence of *Helicobacter pylori* infection in subtypes of gastric cancer. *Gastroenterology* **109**: 885–888

Hennessy TP (1996) Multimodal therapy for carcinoma of the cardia. *Dis Esophagus* **9**: 187–190

Holscher AH, Bollschweiler E and Siewert JR (1995) Carcinoma of the gastric cardia. *Ann Chir Gynaecol* **84**: 185–192

Kalish RJ, Clancy PE, Orringer MB and Appelman HD (1984) Clinical, epidemiologic, and morphologic comparison between adenocarcinomas arising in Barrett's oesophageal mucosa and in the gastric cardia. *Gastroenterology* **86**: 461–467

Locke GR, Talley NJ, Carpenter HA, Harmsen WS, Zinsmeister AR and Melton JL (1995) Changes in the site- and histology-specific incidence of gastric cancer during a 50-year period. *Gastroenterology* **109**: 1750–1756

Loof L, Gottell P and Elfberg B (1993) The incidence of reflux oesophagitis. *Scand J Gastroenterol* **28**: 113–118

McKinney PA, Sharp L, MacFarlane GJ and Muir CS (1995) Oesophageal and gastric cancer in Scotland 1960–90. *Br J Cancer* **71**: 411–415

Miwa K, Sahara H, Segawa M, Kinami S, Sato T, Miyazaki I and Hattori T (1996) Reflux of duodenal and gastroduodenal contents induces oesophageal carcinoma in rats. *Int J Cancer* **67**: 269–174

Percy C, Holten VV and Muir C (1990) *International Classification of Diseases for Oncology*. World Health Organization: Geneva

Powell J and McConkey CC (1990) Increasing incidence of adenocarcinoma of the gastric cardia and adjacent sites. *Br J Cancer* **62**: 440–443

Ricaurte O, Flejou JF, Vissuzaine C, Goldfain D, Rotenburg D, Cadiot G and Potet P (1996) *Helicobacter pylori* infection in patients with Barrett's oesophagus: a prospective immunohistochemical study. *J Clin Pathol* **49**: 176–177

Riddell RH (1996) The biopsy diagnosis of gastroesophageal reflux disease, 'carditis', and Barrett's esophagus, and sequelae of therapy. *Am J Surg Pathol* **20**: S31–S50

Siewert JR and Stein HJ (1996) Carcinoma of the gastroesophageal junction-classification, pathology and extent of resection. *Dis Esophagus* **9**: 173–182

Siewert JR and Stein HJ (1998) Classification of adenocarcinoma of the oesophagogastric junction. *Br J Surg* **85**: 1457–1459

Sobin LH and Wittekind Ch (Eds) (1997) *TNM Classification of Malignant Tumours* pp. 8, 54–55, 59. Wiley: New York

Spechler SJ and Goyal RK (1986) Barrett's esophagus. *N Engl J Med* **315**: 362–371

Spiessl B, Beahrs OH, Hermanek P, Hutter RVP, Scheibe O, Sobin LH and Wagner G (Eds) (1992) *TNM Atlas*: *Illustrated Guide to the TNM/pTNM Classification of Malignant Tumours*, pp. 52–53. Springer-Verlag: Berlin

Stipa F, Ferri M, Aromatorio C and Stipa S (1996) Is there a place for proximal gastric resection in carcinoma of the cardia? *Dis Esophagus* **9**: 183–186

Stipa S, Di Giorgio A and Ferri M (1992) Surgical treatment of adenocarcinoma of the cardia. *Surgery* **111**: 386–393

Tachimori Y, Kato H, Watanabe H, Sasako M, Kimoshita T and Morayama K (1996) Difference between carcinoma of the lower oesophagus and the cardia. *World J Surg* **20**: 507–511

Thomas RJS, Lade S, Giles GG and Thursfield V (1996) Incidence trends in oesophageal and proximal gastric carcinoma in Victoria. *Aust N Z Surg* **66**: 271–275

Thomas RM and Sobin LH (1995) Gastrointestinal cancer. *Cancer* **75**: 154–170

Wang HH, Antonioli DA and Goldman H (1986) Comparative features of oesophageal and gastric adenocarcinomas: recent changes in type and frequency. *Hum Pathol* **17**: 482–487

Whelan SL and Young J (1997) Classification and coding. In *Cancer Incidence in Five Continents*, Vol. VII. Parkin DM, Whelan SL, Ferlay J, Raymond L and Young J (Eds) IARC Scientific Publications 143: Lyon

 *© Cancer Research Campaign 1999*
